# Comparative genomics reveals intraspecific divergence of *Acidithiobacillus ferrooxidans*: insights from evolutionary adaptation

**DOI:** 10.1099/mgen.0.001038

**Published:** 2023-06-07

**Authors:** Rui Liu, Liyuan Ma, Hongmei Wang, Deng Liu, Xiaolu Lu, Xinping Huang, Shanshan Huang, Xueduan Liu

**Affiliations:** ^1^​ Hubei Key Laboratory of Yangtze Catchment Environmental Aquatic Science, School of Environmental Studies, China University of Geosciences, Wuhan 430074, PR China; ^2^​ Hubei Key Laboratory of Wetland Evolution & Ecological Restoration, School of Environmental Studies, China University of Geosciences, Wuhan 430074, PR China; ^3^​ School of Engineering, Cardiff University, Cardiff CF243AA, UK; ^4^​ State Key Laboratory of Biogeology and Environmental Geology, China University of Geosciences, Wuhan 430074, PR China; ^5^​ School of Minerals Processing and Bioengineering, Central South University, Changsha 410083, PR China

**Keywords:** *Acidithiobacillus ferrooxidans*, comparative genomics, evolutionary process, intraspecific divergence, environmental adaptation

## Abstract

*
Acidithiobacillus ferrooxidans* serves as a model chemolithoautotrophic organism in extremely acidic environments, which has attracted much attention due to its unique metabolism and strong adaptability. However, little was known about the divergences along the evolutionary process based on whole genomes. Herein, we isolated six strains of *A. ferrooxidans* from mining areas in China and Zambia, and used comparative genomics to investigate the intra-species divergences. The results indicated that *A. ferrooxidans* diverged into three groups from a common ancestor, and the pan-genome is ‘open’. The ancestral reconstruction of *A. ferrooxidans* indicated that genome sizes experienced a trend of increase in the very earliest days before a decreasing tendency during the evolutionary process, suggesting that both gene gain and gene loss played crucial roles in *A. ferrooxidans* genome flexibility. Meanwhile, 23 single-copy orthologous groups (OGs) were under positive selection. The differences of rusticyanin (Rus) sequences (the key protein in the iron oxidation pathway) and type IV secretion system (T4SS) composition in the *A. ferrooxidans* were both related to their group divergences, which contributed to their intraspecific diversity. This study improved our understanding of the divergent evolution and environmental adaptation of *A. ferrooxidans* at the genome level in extreme conditions, which provided theoretical support for the survival mechanism of living creatures at the extreme.

## Data Summary

All sequenced genomes from this study are available from GenBank through Bioproject PRJNA863019 (accession numbers Biosample SAMN30006355-SAMN30006360). The Whole Genome Shotgun projects of six newly sequenced *A. ferrooxidans* have been deposited at DDBJ/ENA/GenBank under the accession numbers JANJGT000000000 (GD-0), JANJOZ000000000 (GD-A), JANJPA000000000 (GD-B), JANJPB000000000 (ZBY), JANJPC000000000 (BN), JANJPD000000000 (DX). The versions described in this paper are version JANJGT010000000, JANJOZ010000000, JANJPA010000000, JANJPB010000000, JANJPC010000000, JANJPD010000000, respectively. All GenBank accession numbers of the genomes used in this study are provided in Table 1.

**Impact Statement**

*
A. ferrooxidans *, the microorganisms that thrive in extremely acid environments, is a key model for research on biological adaption. The study on the extremophiles could provide hints for the origin and evolution of life, as well as improve the understanding of biogeochemical cycling of elements. Here, we isolated and sequenced six *A. ferrooxidans* strains, and performed comparative genomic analyses to investigate the intra-species divergences. It was implicated that *A. ferrooxidans* diverged into three groups from a common ancestor. Additionally, gene content variation drove adaptive evolution of the genomes, and metabolism-related OGs were more subject to positive selection. This study provided evidences for the relatedness between the hereditary variation of *A. ferrooxidans* genomes with their adaptive evolution, and advanced our understanding of evolutionary strategies of *A. ferrooxidans* genomes.

## Introduction

Extremophiles are specifically adapted to their particular niche environments, and play crucial roles in the cycles of geochemical elements. In acid mine drainage (AMD) with high concentrations of heavy metals and low pH (pH <3), the survival strategy and environmental adaptation mechanism of microorganisms inhabiting here have become one of the research hotspots. *Acidithiobacillus* (formerly *Thiobacillus *) are Gram-negative, obligate autotrophic bacteria, which is commonly observed along with *Acidiphilium* and *Leptospirillum* in AMD [1]. A previous study divided *Acidithiobacillus* into five clades based on 16S rRNA: *A. caldus *, *A. ferrooxidans *, and *A. ferridurans* occupying clades I, II and V, respectively; *A. ferrivorans* and *A. ferriphilus* occupying clade IV, and *A. thiooxidans* and *A. albertensis* occupying clade III [2]. There were also phylogenetic trees constructed with *Acidithiobacillus* conserved proteins, which were divided into 10 clades [3]. To date, the genus *Acidithiobacillus* comprises nine validly published species, including the main *A. ferrooxidans *, *A. thiooxidans *, *A. caldus* and *A. ferrivorans* [4]. *A. ferrooxidans* is the earliest discovered species within *Acidithiobacillus *, which gains energy by utilizing ferrous iron and reduced sulphur compounds as electron donors in the presence of oxygen, or obtains energy by using ferric iron and oxidized sulphur as an electron acceptor via anaerobic metabolisms [5]. From a fundamental perspective, *A. ferrooxidans* are model bacteria to study not only the chemolithoautotrophic energy conversion and pathway, but also the evolutionary adaptation under acidic conditions [6].

With the rapid development of high throughput sequencing technologies and the continuous update of bioinformatics analysis methods, the whole-genome sequencing data has increased significantly, leading to a new research field of comparative genomics. It is an effective way to dig the bacterial evolutionary and genetic information from pan-genome site. The genomes involved in pan-genomics are regarded as a whole and divided into core genes, accessory genes and unique genes. Both accessory and unique genes were categorized as dispensable genomes [7]. The genetic traits concerning adaptation, resistance and virulence were more often governed by dispensable genomes [8]. Moreover, the dispensable genome was traditionally recognized to be responsible for species diversity and probably contributed to selective advantages [2]. Previous studies primarily focused on the metabolic diversity and gene functions of strains at the genome level to explore their adaptation abilities as well as improve the implication in bioleaching, including the central carbon metabolic pathways and the oxidation of iron and reduced inorganic sulphur compounds (RISCs) [9–12]. In addition, due to the extremely acidic environment that is often accompanied by the high concentrations of heavy metals, many considerable efforts have been devoted to the heavy metal resistance of *Acidithiobacillus* to better understand their strategies against toxic metals, such as motility, adhesion and biofilm formation [13, 14]. Moreover, horizontal gene transfer (HGT), natural selection and gene duplication are three main engines that drive the adaptive evolution of microbial genomes [15, 16]. HGT usually occurs through mobile genetic elements (MGEs) carrying functional genes, which helps acidophiles improve their adaptability during evolution [17].

Kimura Motoo’s neutral theory of evolution [18] acknowledged that biological phenotypes evolved under natural selection, meanwhile emphasizing the role of genetic drift. When the mutations are beneficial and can endow with adaptive advantages, these genes may be under diversified positive selection. Positive selection can mediate survival fitness by adaptive mutations, and has also been an indispensable driving force in microbial evolution [19, 20]. In contrast, most non-synonymous substitutions are harmful to individuals during the evolutionary process, because a small change in a functional protein may have lethal effects on organisms. Generally, the mutations of these related genes are easily eliminated by purification (negative) selection [13]. If genes become useless in a population with no selection pressure, they are in neutral evolution, and these genes are more likely to be lost during evolution [21]. The dN/dS ratio can be used to determine whether the gene is under positive selection, which mainly shows dN/dS>1. Genome streamlining is also an adaptive strategy for extremophiles to cope with severe environments [22]. Previous study has shown that the genomes of *Acidithiobacillus* in hot springs in the Taupo Volcanic Zone (TVZ) of New Zealand are usually smaller, and it was found that broadly lower proportions of non-coding RNA to estimated genome size in TVZ *Acidithiobacillus* spp. [23]. These characteristics of genome streamlining are all associated with the adaptation of strains to high temperature environments [24].

**Fig. 1.**
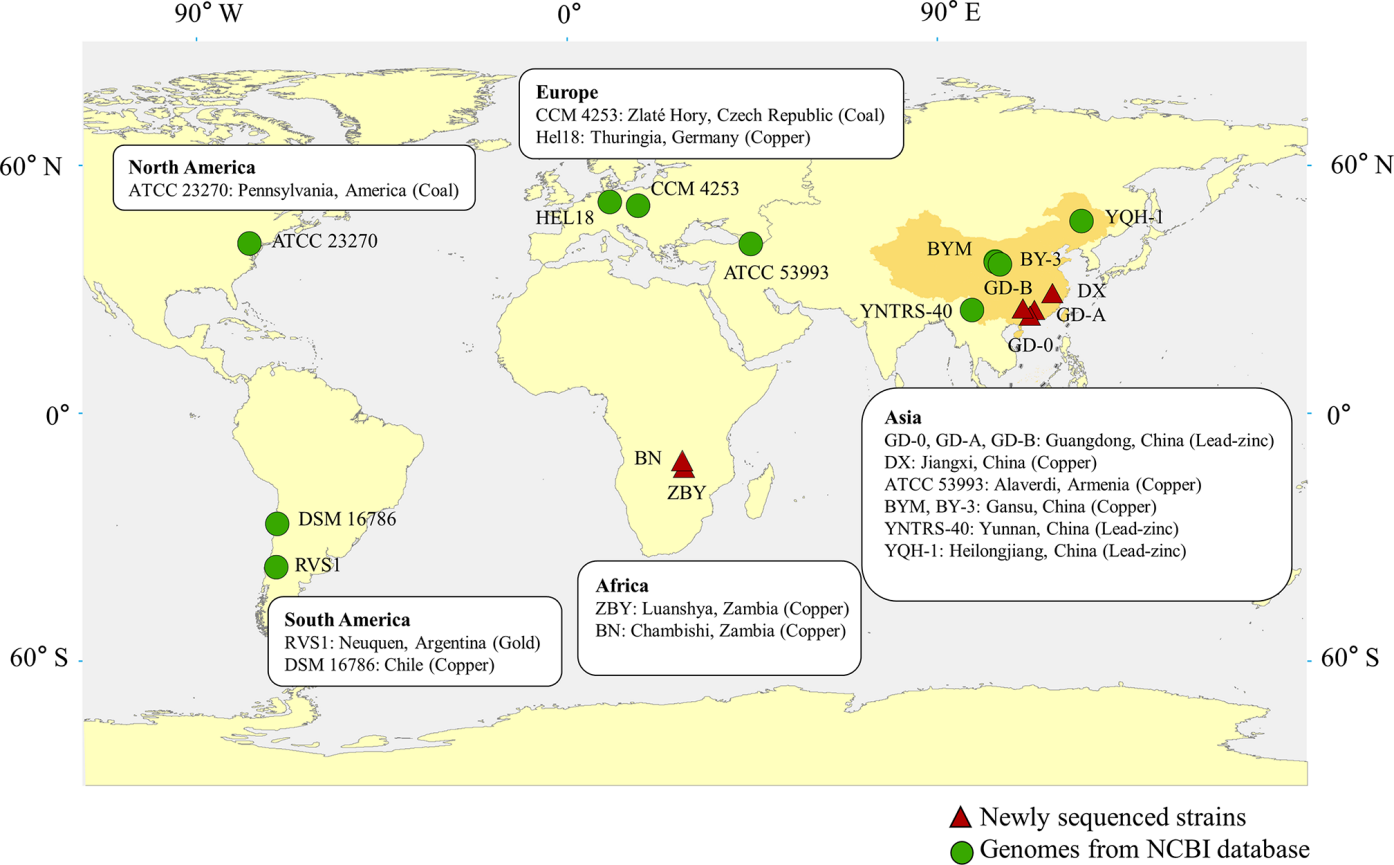
Geographic locations of 16 *A *. *ferrooxidans* strains, including the ore type where the samples were collected.

Although there have been some reports on the metabolic potential and adaptive mechanism of the genus *Acidithiobacillus* [14, 25, 26], how they diverged during the evolutionary process in extreme environments still remains unclear. To unravel their evolutionary history and assess the divergences in metabolic and niche adaption, we isolated and sequenced six strains within the genus *Acidithiobacillus* from copper mines of China and Zambia to do comparative genomic analyses together with the available genomes in the public database (Fig. 1). The six newly sequenced strains were divided into three groups belonging to *A. ferrooxidans* species according to their phylogeny analysis. The gene content evolution and the positive selection were analysed by evaluating the orthologous groups (OGs) of *A. ferrooxidans *. The aims of this study are (i) How do the intraspecific divergences of *A. ferrooxidans* arise during the evolutionary process? (ii) How does these intraspecific divergences contribute to *A. ferrooxidans* adaptation to extreme environments? These results will provide insights into the survival mechanisms of extremophiles.

## Methods

### Bacterial genomes used in this study

We isolated six *Acidithiobacillus* spp., including strains GD-A, GD-B and GD-0 from acid mine drainage, Shaoguan copper mine of Guangdong Province (China), strain DX from Dexing copper mine of Jiangxi Province (China), strain ZBY from Luanshya copper mine (Zambia), and strain BN from a heap leaching plant of Chambishi copper mine (Zambia). More details for the geographic origins of these strains are shown in Fig. 1. The six *A. ferrooxidans* strains were cultivated using the typical 9 K medium [(NH4)_2_SO_4_ 3.0 g l^–1^, KCl 0.1 g l^–1^, K_2_HPO_4_ 0.5 g l^–1^, MgSO_4_⋅7H_2_O 0.5 g l^–1^, Ca(NO_3_)_2_ 0.01 g l^–1^] with FeSO_4_⋅7H_2_O (30.0 g l^–1^) and pH adjusted to 2.0 under the temperature of 30 °C. Then they were incubated aseptically in a shaker at 170 rpm.

Genomic DNA was extracted and purified from the washed cells using TIANamp Bacteria DNA kit (TIANGEN, Beijing, China). The shotgun library was constructed with an average DNA insert size of 300 bp, and then, the DNA library was sequenced by the Illumina MiSeq sequencing platform (Illumina, California, USA) using paired-end sequencing approaches. The raw reads were assembled into contigs using the SOAPdenovo2 package [27]. In addition, 21 genomes of seven species within *Acidithiobacillus* available in the National Centre for Biotechnology Information (NCBI) repository were collected, based on the following screening criteria: all the available *A. albertensis *, *A. ferrianus* and *A. sulfuriphilus* before 22 October 2021 were selected. Meantime, the *A. ferrooxidans *, *A. ferrivorans *, *A. ferridurans* and *A. ferriphilus* with contigs<200 were picked out.

CheckM was used to assess the quality of each genome [28]. The identification of transfer RNA (tRNA) and ribosomal RNA (rRNA) were performed using tRNAscan-SE v2.0 (http://lowelab.ucsc.edu/tRNAscan-SE) [29] and RNAmmer v1.2 [30] programmes. Then, a 16S rRNA sequence-based phylogenetic tree was analysed using IQ-Tree v 1.6.12 [31] with automatic choice of the best-fit model (-m MFP) using maximum likelihood (ML) method. Ultrafast bootstrap support values were calculated from 1,000 replicates (-bb 1000 -bnni). The tree was visualized and further beautified through the online server iTOL (https://itol.embl.de) [32]. A genome-based phylogeny of *Acidithiobacillus* spp. was constructed using the online platform CVTree4 with K-tuple length 9 (cvtree.online/v4/prok/index.html) [33]. The average nucleotide identity (ANI) based on blast algorithm (ANIb) and MUMmer algorithm (ANIm), as well as tetranucleotide frequency correlation coefficient (TETRA), were further calculated using the web server JSpeciesWS (https://jspecies.ribohost.com/jspeciesws/) [34]. R v3.6.3 with RStudio v1.2.5001 software, and R package pheatmap were used for visualization. In order to further investigate the taxonomic status of *A. ferrooxidans* among acidic chemolithoautotrophs, a phylogenetic tree of acidophilic chemoautotrophic microorganisms including 80 bacteria and archaea was constructed using the same method as the above.

The coding DNA sequences (CDSs) of the six newly sequenced strains were identified using the PROkaryotic DYnamic programming Gene-finding Algorithm (prodigal v2.6.3. -p single) [35]. Then CDSs were annotated by blast searches against the bacteria databases of NCBI RefSeq non-redundant (NCBI-nr) proteins and Clusters of Orthologous Groups (COG) [36] with an *E*-value to the threshold of 1e^−5^.

### Pan-genome analysis

Based on the phylogenetic relationship of six sequenced *Acidithiobacillus* strains, 10 genomes of *A. ferrooxidans* were selected for further comparative analysis (Fig. 1). The Bacterial Pan-genome Analysis tool (BPGA v1.3) was used to classify orthologous into core, accessory and unique genomes. According to the results, the strains with relatively more unique genes were annotated using the blast algorithm against the COG database. Then, functional classifications of core, accessory and unique genes were performed using blast algorithm against the COG and Kyoto Encyclopaedia of Genes and Genomes (KEGG) database [37]. USEARCH v11.0.667 programme available in BPGA was performed to estimate the pan-genome and core genome, with a 50 % cut-off of sequence identity [38]. The nonlinear fitting was performed based on the model extrapolation of the pan-genome and core genome.

### Gene content evolution of *A. ferrooxidans*

OrthoFinder v2.3.12 was used to cluster the protein sequences of *A. ferrooxidans* into OGs (with default parameters) [39]. Single-copy gene tree were constructed through the EasySpeciesTree v1.0 script using the ML method of RAxML v8.0.26. To explore the gene content evolution of *A. ferrooxidans *, ancestral gene numbers were inferred using the programme COUNT v9.1106 [40] with Dollo parsimony. This approach strictly prohibits multiple gains of genes and allows reconstructing gene gain and loss events at both observed species and potential ancestors (leaves and nodes on the phylogenetic tree). The gain and loss genes of several key nodes were annotated through the COG database.

### Positive selection analysis

The single-copy OGs sequences alignment was carried out by clustalw v2.1 [41], and the required file format was completed by ParaAT v1.0 [42]. The non-synonymous and synonymous substitution (dN/dS ratio) of each single-copy OG were applied by PAML4 codeml programme [43]. Furthermore, the OGs of dN/dS ratio >1 were assigned into COG categories through blastp.

### Rusticyanin (Rus) and type IV secretion system (T4SS) analysis

The multiple amino acid sequence alignments of Rus (encoded by *rus*), which is a key enzyme involved in the iron oxidation pathway, were completed by Jalview v2.11.2.2 [44] using muscle [45], then the similarities and differences between them were analysed. The genes related to T4SS, a common secretory system in Gram-negative and Gram-positive bacteria, were identified in *A. ferrooxidans* strains by the same method with Rus and displayed by heatmap using the online server ChiPlot (https://www.chiplot.online/).

## Results and discussion

### General genomic features and phylogenetic analysis of six newly sequenced strains

The genomic features of the newly sequenced *A. ferrooxidans* are summarized in Table 1. All genomes completeness involved in this study were 100 %, and contamination were 0 %, which ensured the credibility of the further analysis. Generally, the genome lengths varied from 2 771 628 to 3 411 247 bp, accompanied by the CDSs number ranging from 2873 to 3671. The number of predicted tRNAs, which encoded almost all 20 amino acids, ranged from 48 to 90. Moreover, DX, ZBY and BN have larger genomes, but their GC content was slightly lower than the counterparts. Similar observations have been documented in *Leptospirillum ferriphilum* Sp-Cl, whose genome was larger but GC content was lower when compared with other *L. ferriphilum* [46]. The three hydrogen bonds bound by GC confer higher thermal stability on DNA than the two hydrogen bonds bound by AT [47].

**Table 1.** General features of *A. ferrooxidans* genomes used in this study, including six newly sequenced strains and 10 strains available on NCBI

Strain	Genome status	Accession no.	Completeness (%)	Contamination (%)	Total bases (bp)	GC (%)	Contigs	Coverage	N50 (bp)	Max (bp)	Min (bp)	CDSs (Total)	rRNAs	tRNA
GD-0	Draft	JANJGT000000000	100.00	0.00	2771628	58.9	139	127.0 x	35 716	108 579	585	2873	1, 1, 1(5S, 16S, 23S)	48
GD-A	Draft	JANJOZ000000000	100.00	0.00	2786301	58.9	135	136.3 x	57 501	121 067	504	2879	1, 1, 0(5S, 16S, 23S)	48
GD-B	Draft	JANJPA000000000	100.00	0.00	2795191	58.9	155	166.3 x	39 339	130 501	502	2910	2, 1, 1(5S, 16S, 23S)	48
ZBY	Draft	JANJPB000000000	100.00	0.00	3400916	58.5	159	160.7 x	42 734	135 905	504	3647	1, 1, 0(5S, 16S, 23S)	90
BN	Draft	JANJPC000000000	100.00	0.00	3411247	58.5	171	132.6 x	47 044	179 645	504	3671	1, 1, 1(5S, 16S, 23S)	90
DX	Draft	JANJPD000000000	100.00	0.00	3 158 850	58.5	201	189.3 x	30 087	130 503	514	3355	1, 1, 1(5S, 16S, 23S)	53
ATCC 23270	Complete	NC_011761	100.00	0.00	2 982 397	58.8	1	nd	2 982 397	2 982 397	2 982 397	2983	2, 2, 2(5S, 16S, 23S)	82
ATCC 53993	Complete	NC_011206	100.00	0.00	2 885 038	58.9	1	nd	2 885 038	2 885 038	2 885 038	2862	2, 2, 2(5S, 16S, 23S)	46
DSM 16786	Draft	NZ_JABFOH010000000	100.00	0.00	3 675 789	58.4	49	124.0 x	292 477	810 280	1031	3782	2, 2, 2(5S, 16S, 23S)	82
BYM	Complete	NZ_CP082238	100.00	0.00	3 208 389	58.5	1	352.0 x	3 208 389	3 208 389	3 208 389	3262	2, 2, 2(5S, 16S, 23S)	47
CCM 4253	Draft	NZ_QKQP01000000	100.00	0.00	3 196 562	58.6	15	496.64 x	683 820	1 474 269	516	3194	2, 2, 2(5S, 16S, 23S)	47
BY-3	Draft	NZ_AZNR01000000	100.00	0.00	3 832 339	57.8	194	211.5 x	76 215	296 345	308	4005	1, 1, 1(5S, 16S, 23S)	73
Hel 18	Draft	NZ_LQRJ01000000	100.00	0.00	3 109 160	58.6	123	1.0 x	59 423	222 655	495	3148	1, 1, 1(5S, 16S, 23S)	46
YNTRS-40	Complete	NZ_CP040511	100.00	0.00	3 209 933	58.5	1	152.25 x	3 209 933	3 209 933	3 209 933	3255	2, 2, 2(5S, 16S, 23S)	47
YQH-1	Draft	NZ_LJBT01000000	100.00	0.00	3 109 477	58.6	96	285.0 x	69 681	244 797	1000	3152	2, 3, 4(5S, 16S, 23S)	45
RVS1	Draft	RRZY01000000	100.00	0.00	2 826 311	58.8	49	125.0 x	241 921	465 740	216	2832	2, 2, 2(5S, 16S, 23S)	46

**Fig. 2.**
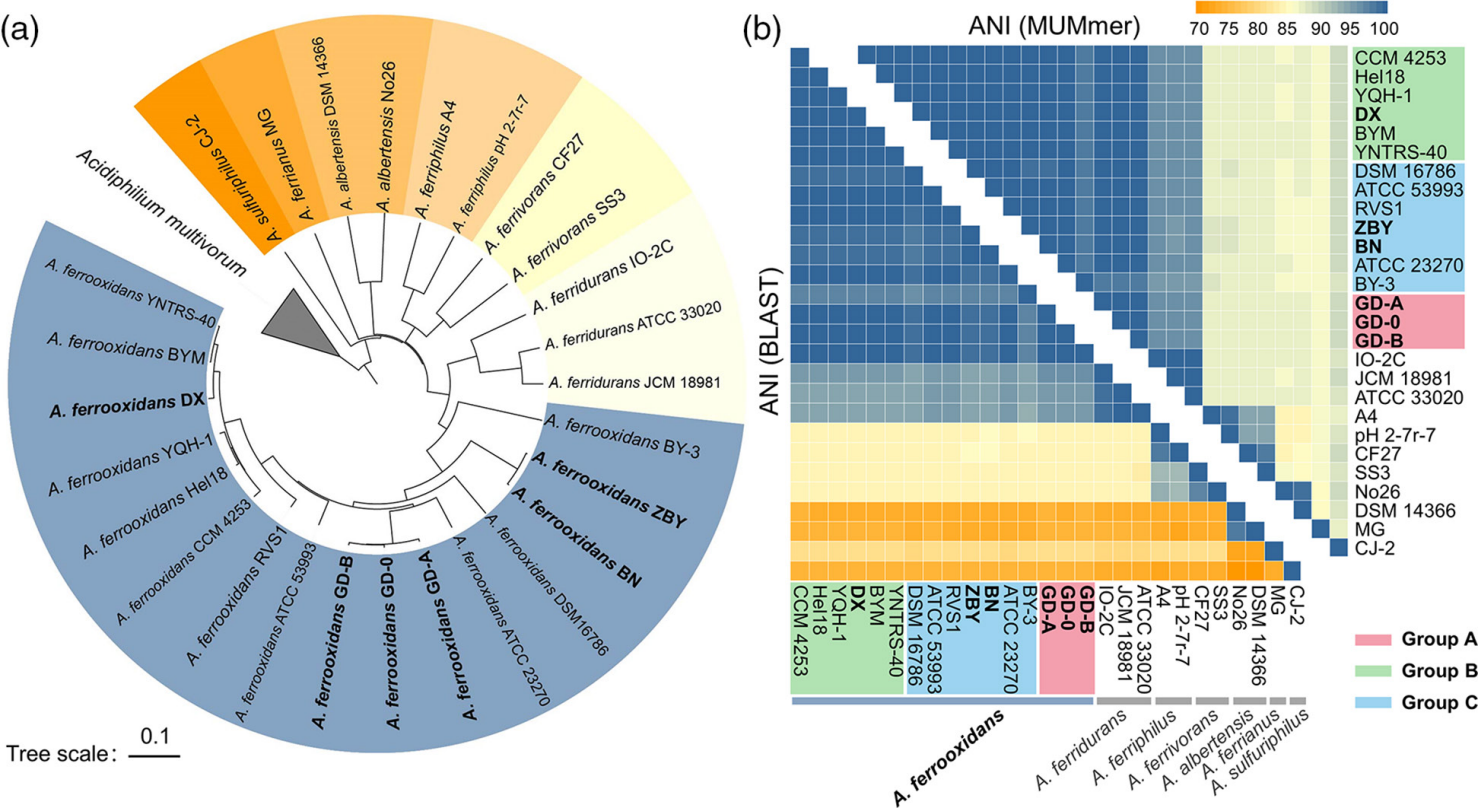
Determination of the taxonomic status of six newly sequenced *Acidithiobacillus* strains. (**a**) Phylogenetic tree of whole genomes using CVTree4 (k=9) showing the relationship between six sequenced strains and other *Acidithiobacillus* strains from NCBI. *Acidiphilium multivorum* was the outgroup. The newly sequenced strains were thickened, and the 16 *A *. *ferrooxidans* strains involved in this study were divided into three groups according to their relationship: GD-0, GD-A and GD-B were in group A, DX was in group B, ZBY and BN were in group C. (**b**) The heat map of ANI values, using both blast and MUMmer methods, detailed values referred to Table S1. The higher ANI value, the closer the genetic relationship between the strains. The cut-off value proposed for species distinction is about 95–96 %.

In the 16S rRNA gene-based phylogenomic tree, the six newly sequenced strains were clustered together, which was apparently away from other known *Acidithiobacillus* spp., such as *A. ferrivorans *, *A. ferriphilus *, *A. albertensis* and *A. ferridurans* (Fig. S1, available in the online version of this article). Meantime, the phylogenetic tree based on whole-genome sequences was constructed to assess the relationships among the newly sequenced strains and other species within the genus *Acidithiobacillus* (Fig. 2a). The dendrogram showed that all the newly sequenced strains were also distinguished from other *Acidithiobacillus* isolates at genome level. The strains GD-A, GD-B and GD-0, which were isolated from Fankou lead-zinc mine, were clustered on a distinct branch, namely Group A. The strain DX was closely related to the nearest BYM and YNTRS-40, was classified in Group B. Meanwhile, the other two strains, BN and ZBY, which were originated from copper mines of Zambia, occupied the same clade and were assigned into Group C. The results indicated that the genomic differentiation of the six *A. ferrooxidans* strains were closely related to their geographic locations. ANIb, ANIm and TETRA were further calculated to infer the phylogenetic relationships among *Acidithiobacillus* strains (Fig. 2b and Table S1). The six newly sequenced strains and *A. ferrooxidans* had high values of ANIb (≥95.76 %), ANIm (≥97.85 %) and TETRA (≥0.997), above the cut-off value (ANI ~95–96 % and TETRA ~0.99) [48], which indicated that the newly sequenced strains were very closed related to all known *A. ferrooxidans* strains (Table S1). The phylogenetic tree (Fig. S2) based on 16S rRNA sequences of the acidic chemolithoautotrophs showed that *Gammaproteobacteria *, which *A. ferrooxidans* belong to, were located in the same branch with *Betaproteobacteria *, as similar to the result of a previous study [49].

Of all isolates, functional analysis based on COG classification revealed that the three most abundant function categories were M (cell wall/membrane/envelope biogenesis, 6.75–7.54 %), J (translation, ribosomal structure, and biogenesis, 5.43–6.77 %) and C (energy production and conversion, 5.31–6.09 %) (Fig. S3 and Table S2). M and C were also the most abundant categories in the genomes of *A. caldus* based on COG classification [50]. In addition, the abundant function categories in *Leptospirillum ferriphilum* also included M, C and J [46]. These COG categories of CDSs have been reported to be necessary for acid and heavy metal resistance and, likely, long-term adaptation mechanisms to the extreme environments [51, 52]. It may be the common species in AMD environments shared similar major metabolic functions and evolutionary approaches.

### Pan-genome analysis of *A. ferrooxidans* species

**Fig. 3.**
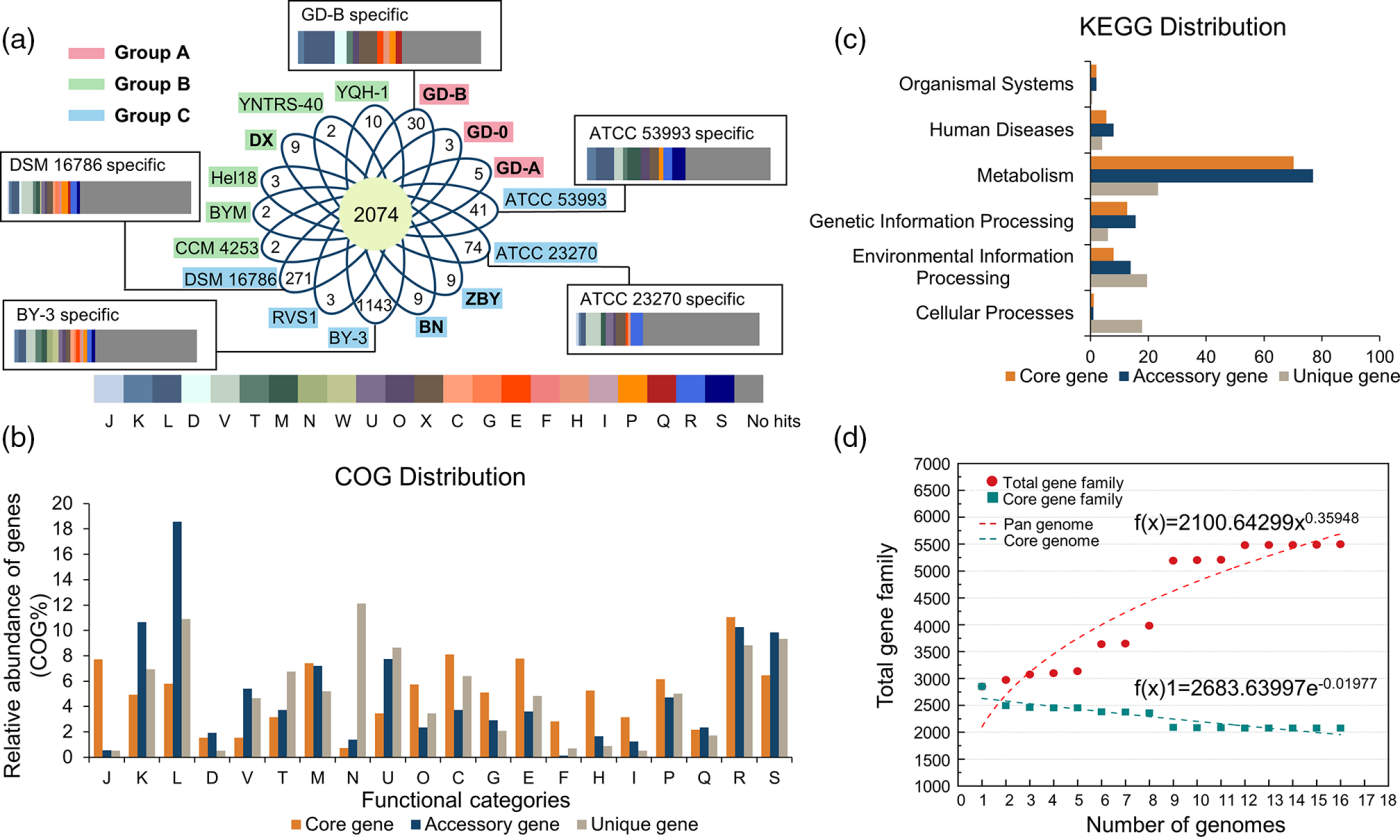
Pan-genome analysis of 16 *A *. *ferrooxidans* strains. (**a**) Flower plot showing the number corresponding to the unique genes (in the petals) of each strain, and the number of core genes common to all strains (in the centre), Bar stacking diagram showing that COG classification about unique genes. Abbreviations: J, translation, ribosomal structure, and biogenesis; K, transcription; L, replication, recombination, and repair; D, cell cycle control, cell division, chromosome partitioning; V, defence mechanisms; T, signal transduction mechanisms; M, cell wall/membrane/envelope biogenesis; N, cell motility; W, extracellular structures; O, post-translational modification, protein turnover and chaperones; U, intracellular trafficking, secretion, and vesicular transport; X, mobilome: prophages, transposons; C, energy production and conversion; G, carbohydrate transport and metabolism; E, amino acid transport and metabolism; F, nucleotide transport and metabolism; H, coenzyme transport and metabolism; I, lipid transport and metabolism; Q, secondary metabolites biosynthesis, transport and catabolism; P, inorganic ion transport and metabolism; R, general function prediction only; S, function unknown. (**b**) COG assignments of core, accessory, and unique genes. (**c**) KEGG assignments of core, accessory, and unique genes. (**d**) Mathematical extrapolation for estimating the size of *A. ferrooxidans* pan-genome and core genome.

To understand the pan-genome of *A. ferrooxidans* more deeply, CDSs obtained from the genomes were clustered into 5495 gene families using BPGA. A core genome of 2074 CDSs was obtained, which represented 57–79 % of each genome in the selected population (Fig. 3a). The percentages of core genes in each genome were higher than those in *A. thiooxidans* (52–54.8 %) [53]. Core genome is related to major cellular processes, such as transcription, translation and biosynthesis of nucleotide, lipid, amino acid and carbohydrate, as well as energy production and conversion. These essential genes allow acidophiles to efficiently take up nutrients from the environment, as well as maintain a basic lifestyle. Therefore, the core genome facilitates the persistence and proliferation of *A. ferrooxidans* in the harsh habitat [8]. Meantime, the number of unique genes in *A. ferrooxidans* genomes varied from 2 to 1143, implying the individual differences and a relatively high degree of genomic diversity. More unique genes were observed in Group C, indicating that they may adapt to the extreme environments by diverse strategies (Fig. 3a and Table S3). BY-3 had the largest number of unique genes, no matter compared with all the selected strains or with ATCC 23270 and ATCC 53993 (Fig. S4A). Focusing on the six newly sequenced strains, we found that DX harboured the most unique genes while others maintained very few specific genes (Figs S4B and S4C). These results suggested a close correlation of the newly sequenced strains within *A. ferrooxidans *.

The core, accessory and unique genes were annotated through COG and KEGG databases in BPGA (Fig. 3b, c). The core genome harboured the highest number of basic metabolic profiles, especially COG categories C (energy production and conversion, 8.1 %) and E (amino acid transport and metabolism, 7.8 %), suggesting the core genes encoded biological functions that were essential to basic lifestyles and phenotypes [46, 54]. However, most accessory genes were related to information storage and processing, such as COG category L (replication, recombination and repair, 18.6 %), suggesting that the acidophiles could self-repair the DNA damage caused by the harsh environment [55]. The genes related to category N (cell motility, 12.1 %) accounted for the highest proportion among unique genes, which could help microorganisms escape from harmful interferences [20]. In addition, similar results were also found in the KEGG classification (Fig. 3c). The majority of genes that were essential to the basic lifestyle made up the core genome. Interestingly, a large number of accessory genes existed in the pathway of metabolism, indicating that the accessory genome played a flexible role in the individual adaptation to environments [56, 57].

We used mathematical extrapolation to determine the expansion of *A. ferrooxidans* pan-genome by BPGA (Fig. 3d). The pan-genome of *A. ferrooxidans* harboured 5495 gene families, fitted into an empirical power law regression function through the Allometric1 model [f(x)= 2100.64299x^0.35948^] with a parameter of 0.35948 (Figs 3d and S5). The parameter exponent (0.35948) is between 0 and 1 and close to 0, which meant the pan-genome is ‘open’ [7, 58]. According to Heaps’ law, an ‘open’ pan-genome has a large and undetermined number of additional genes, and its size would increase unboundedly with the number of strains [59]. In this study, at least two additional strain-specific genes would appear when adding a newly sequenced genome, leading to an ‘open’ pan-genome [46]. In addition, the number of core genes decayed with the genome addition, fitted into an exponential regression through Exp2PMod1 model [f(x)1=2683.63997e^-0.01977x^]. This fitting curve followed a gentle slope, and the number of core genes reached a constant number (2075) when the twelfth genome was added. These results indicated the genome size of *A. ferrooxidans* was not yet saturated.

### Gene content variation drives genome size evolution

**Fig. 4.**
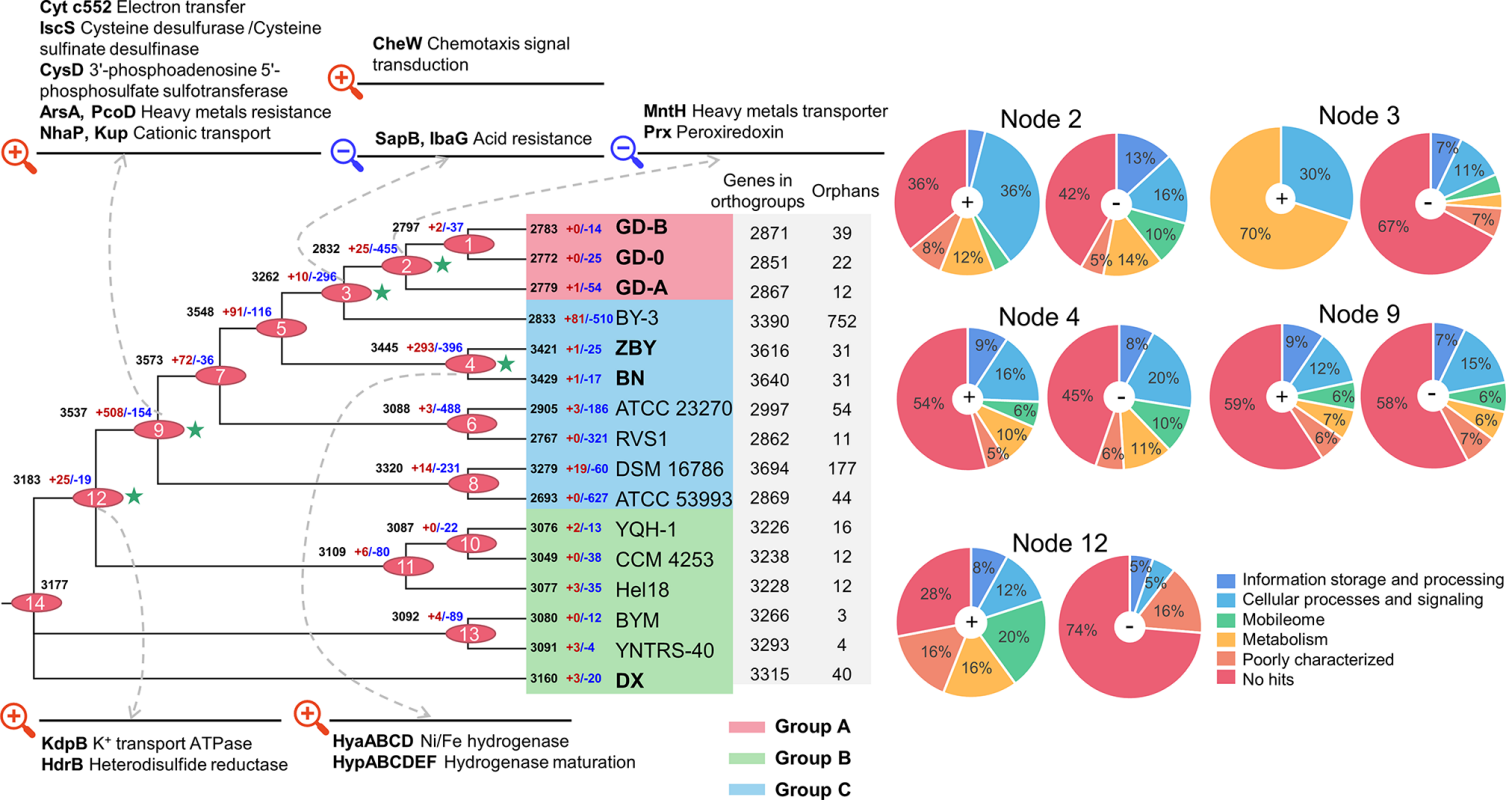
The OrthoFinder was used to find homologous genes of 16 *Acidithiobacillus* strains. Phylogenetic tree constructed by ML method after tandem comparison of 1766 single-copy genes in 16 *Acidithiobacillus* strains. Ancestral genome content reconstruction of the *Acidithiobacillus* genus was performed with Dollo parsimony algorithms implemented in the COUNT software. ‘+” Represented gain events, and ‘-” represented loss events. Numbers on the branches and at the end of branches indicated the number of gain (red) and loss (blue) genes, and the extant counts of genes (black), respectively. COG categories of gain and loss genes at some key nodes were shown in pie charts. Genes involved in the gain and loss events in the *Acidithiobacillus* clades were also labelled at the corresponding nodes.

To explore the divergences of *A. ferrooxidans *, we mapped the parsimony-based gene gain and gene loss events of inferred OGs to 1766 single-copy gene-based phylogenomic trees (Fig. 4). It was inconsistent with the phylogeny based on their whole genomes (Fig. 2a). Generally, housekeeping genes comprised the majority of single-copy datasets, while strain-unique genes were often part of long MGEs that may originate from HGT events [60]. Thus the inconsistency of phylogeny trees may be related to abundant exogenous genes introduced by genetic exchange. The OG number showed a noticeable decrease after an upward trend during the evolutionary process (Fig. 4). A large number of OGs gained in the node 9 (the most recent common ancestor [MRCA] of group A and group C) and 4 (The MRCA of strain ZBY and BN), taking up 14.4 and 8.5 % of OGs at corresponding nodes. Most of the gained/lost genes were involved in cellular processes and signalling (Fig. 4 and Table S4). In addition, many genes related to environmental adaptation were gained at node 12 (Fig. 4, Table S4): as a component of the oligomeric K^+^ transport complex in prokaryotes, KdpB harness ATP energy to drive transport of K^+^ against concentration gradients, which may help *A. ferrooxidans* maintaining cell osmotic pressure in an acidic environment [61]. Additionally, heterodisulphide reductase subunit B (HdrB) was gained at the same node. Hdr is widely found in methanogenic archaea and is involved in carbon dioxide (CO_2_) fixation and methane generation by catalysing the reversible reduction of the heterodisulphide (CoM-S-S-CoB) of the methanogenic thiol coenzymes, coenzyme M (CoM-SH) and coenzyme B (CoB-SH) [62, 63].

Node 9 was the MRCA of group A and C. Cytochrome *552* (cyt *c552*), which was encoded by *cyc1*, was gained at this node. As a part of the *rus* operon, cyt *c552* contributes to transferring electrons to terminal oxidases and plays an important role in iron oxidation [64, 65]. Cysteine desulphurase (IscS) of iron-sulphur cluster (ISC) system was also found at this node. Iron and iron-sulphur protein (HiPIP) may be involved in the electron transport process of the iron oxidation in *A. ferrooxidans* [66], and the synthesis of iron-sulphur cluster in these two enzymes is related to the iron-sulphur proteins in the ISC system. The acquisition of cyt *c552* and IscS were beneficial to the electron transport in iron metabolism. In addition to iron metabolism, 3′-phosphoadenosine 5′-phosphosulphate sulphotransferase (PAPS reductase, CysD), which is related to sulphur metabolism, was obtained at node 9, but this gene was observed to be lost at node 4 and 2 later. CysD is involved in the assimilatory sulphate reduction process from sulphate to sulphite. The presence of CysD could help *A. ferrooxidans* adapt to the AMD environment with high concentration of sulphate. Sulphur oxidation can be considered the most common physiology across the whole class of *Acidithiobacillia *. Previous study found that only *Acidithiobacillus* among *Acidithiobacillia* had the metabolic trait of iron oxidation, supporting the possibility that iron chemolithotrophy might have arrived late in the evolutionary history of the class, which was acquired by HGT from other primitive iron-oxidizing microorganisms [67]. Therefore, *Acidithiobacillus* may have a stronger competitive advantage than other species among *Acidithiobacillia *. Both NhaP and K^+^ uptake protein (Kup) were found at node 9. NhaP-type Na^+^ /H^+^ antiporters are integral membrane proteins, which could transport a wide range of cations to control cellular pH, volume homeostasis and regulate Na^+^/H^+^ levels [68]. Additionally, microorganisms depended on a variety of different K^+^ uptake systems to adapt to rapidly changing external conditions [69]. The acquisition of NhaP and Kup relieved the osmotic stress of the cells. Arsenite/tail-anchored protein-transporting ATPase (*arsA*) gene and copper resistance gene (*pcoD*) were identified at node 9, indicating that the ancestral species of node 9 had the genetic potential to respond to heavy metal stress in the environment [70]. Consequently, we proposed that the environmental condition at node 9 may have changed, and the ancestors acquired multiple adaptabilities to maintain cellular pH and resist heavy metals.

Node 3 was a critical node to differentiate group A strains, and chemotaxis signal transduction protein (CheW) was gained at this node. CheW is a part of the chemoreceptors Methyl-accepting Chemotaxis Proteins (MCPs) in the bacteria chemotaxis system. Chemotaxis offers bacteria the ability to track spatial of chemoeffectors and move towards optimal environments [71]. The acquisition of CheW could allow the strains of group A to better respond to the environmental stress and obtain growth advantages in unfavourable conditions [72]. The gene clusters of Hya and Hyp related to hydrogenase were gained in the MRCA of strain ZBY and BN. Hydrogenase are a group of metal enzymes, such as HyaABCD and HypABCDEF. Many purified hydrogenases have bidirectional catalytic activity, catalysing the oxidation of hydrogen or the reduction of protons [73]. *A. ferrooxidans* may initially rely on the atmospheric energy source as they colonize on barren tailings and establish an acidic microenvironment conducive for iron oxidation [73, 74].

The number of OGs decreased in the nodes ancestral to group A. The ancestors lost large numbers of genes, taking up 9.1 and 16.1 % of OGs at nodes 3 and 2, respectively. Therefore, group A strains harboured smaller genomes. SapB and IbaG were lost at node 3, which has been reported to be related with the microbial acid tolerance [75]. The genes that encoding proteins associated with heavy metal transport were lost at node 2, such as Mn^2+^/Fe^2+^ (MntH) and Cu/Ag (CusA). Meanwhile, the gene encoding peroxiredoxin (Prx) was also lost at node 2, which was relevant to microbial oxidative stress in an AMD environment [76]. The ‘less is more’ hypothesis proposes that gene loss may be an adaptive evolutionary process that is beneficial to organisms [77]. The loss events cause the streamlining of the genomes. To be evolutionarily successful, streamlining requires that the lost functions are dispensable to the organism, such that the cost of gene loss is less than the benefit. Previous studies have shown, for example, that some genes for respiration, catabolic and other pathways are lost in *Mycobacterium leprae *, in which case genes are lost because functions such as host defence or carbohydrate metabolism are no longer needed [78]. Previous studies have shown gene loss as a pervasive source of genetic variation that can cause adaptive phenotypic diversity [79]. Therefore, these lost genes in *A. ferrooxidans* may be the result of adaptive evolution [80].

Additionally, we also calculated the proportions of MGEs in each annotated node (Fig. 4), and these MGEs were mainly transposase and phage-related proteins. MGEs play an essential role in the evolution of bacterial genomes and their resistance to specific environmental stress [81, 82], which suggested gene transfer mediated by MGEs may have given *A. ferrooxidans* a selective advantage in an acidic environment. Both gene gain and gene loss promote environmental adaptation in *A. ferrooxidans *. The gene gain events contributed to the adaptabilities of the acidophiles in an earlier period, while the increase of gene loss events in a later period resulted in the streamlined genome of the strains.

### Positive selection promotes environmental adaptation

**Fig. 5.**
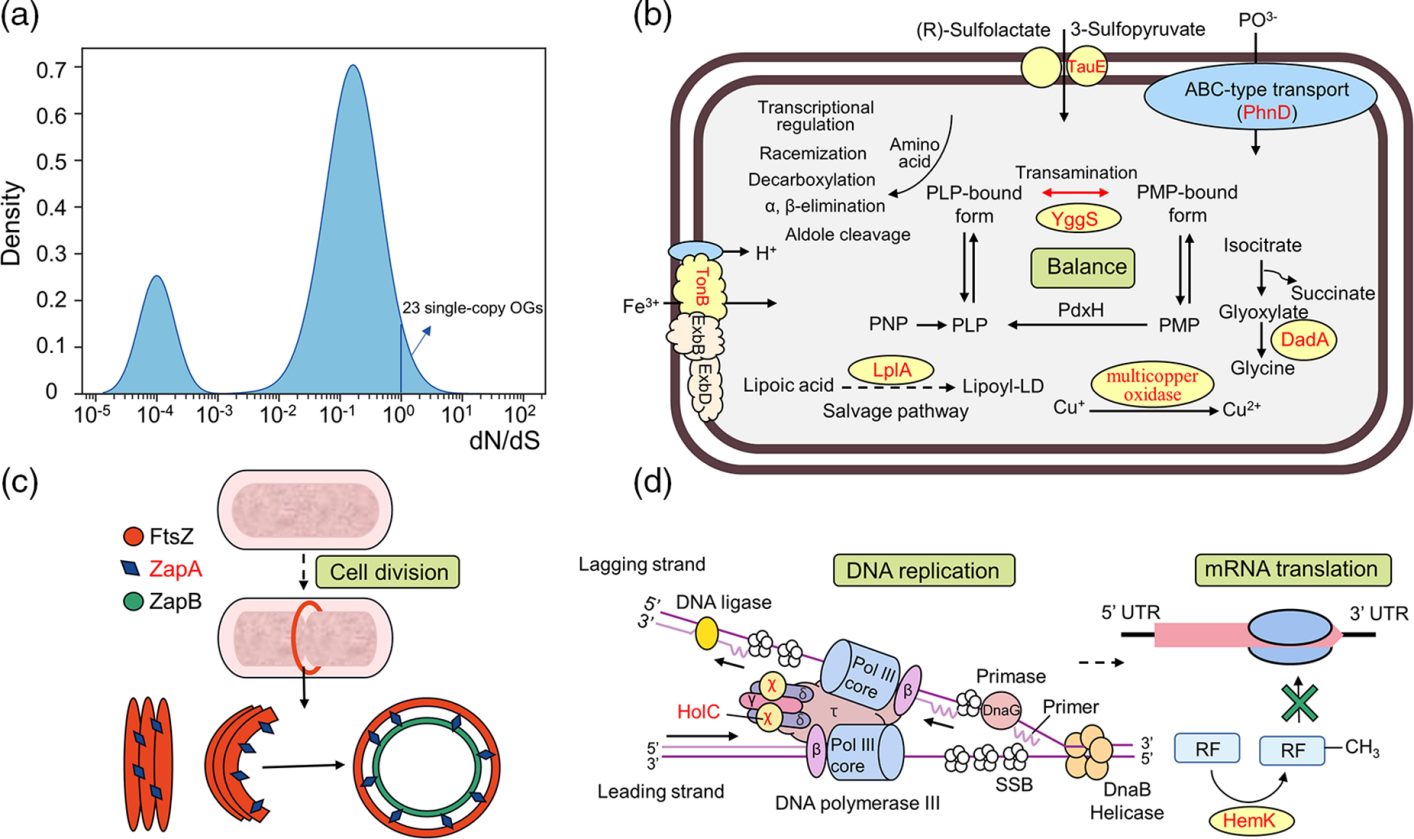
Positive selection analysis. (**a**) Density plot of dN/dS values. PAML was used to calculate the dN/dS values of 1766 single-copy OGs in 16 *A *. *ferrooxidans* strains. Except for the excluded OGs, and a total of 23 groups have a ratio higher than 1. (**b**), (**c**), (**d**) were the process of intracellular metabolism, cell division, DNA replication and translation respectively. Genes marked red were subject to positive selection.

The non-synonymous substitutions rate (dN), the synonymous substitutions rate (dS), and their ratio (dN/dS) are commonly used to aid in understanding the direction of evolution and its selective strength of the protein-coding sequences. The ratio is commonly devoted to identify protein sites that experience purifying selection (dN/dS <1), evolving neutrally (dN/dS ≈ 1), or positive diversifying selection (dN/dS >1) [13, 83]. Previous study focused on those genes under positive selection to reveal the genetic adaptations [84]. We identified 1766 single-copy OGs in the 16 genomes of *A. ferrooxidans *, while 23 single-copy OGs had a dN/dS ratio greater than 1 (Fig. 5a and Table S5).

The result showed that 34.78 % OGs that function under positive selection were related to metabolism, especially coenzyme transport and metabolism (COG category [H]) (Table S5 and Fig. 5b). Specifically, the lipoate-protein ligase A (LplA), which was identified in category [H], was involved in the lipoate biosynthesis by absorbing exogenous lipoic acid in the salvage pathway [85]. YggS was a pyridoxal 5′-phosphate (PLP)-binding protein proposed to be involved in microbial homeostasis [86]. In living cells, PLP as a co-factor for aminotransferases, PLP balance was physiologically indispensable for amino acid metabolism [87]. Sirohydrochlorin ferrochelatase (SirB), a 2Fe-2S protein, catalyses the last step of siroheme biosynthesis utilizing iron [88]. These coenzymes bound specifically to proteins and actively participated in catalytic biotransformation [89], slightly affecting the protein functions to better adapt to the environment by promoting oxidation and reduction at the specific sites.

Also, OGs related to [P] inorganic ion transport and metabolism were identified under positive selection, such as multicopper oxidase, TauE and PhnD (Fig. 5b). The gene encoding multicopper oxidase might be characterized by a close relation to copper tolerance [90]. Previous research showed multicopper oxidase genes involved in copper detoxification were lost in *A. ferrooxidans* and were replaced by the *rus* gene during evolution [13]. Gene *rus* was related to biochemical iron oxidation, which allowed acidophiles to effectively utilize ferrous iron as an energy source under acidic environments [14]. We found sulphite exporter (TauE) was also under positive selection. The chemoautotrophic microorganisms living in AMD obtain energy mainly from iron and/or sulphur oxidation to sustain essential metabolisms such as carbon and nitrogen assimilation [91]. In environments with excessive heavy metal stress, the ABC transporters associated with metal ions and inorganic minerals are frequently detected [70]. Similarly, we also found PhnD, an ABC-type phosphate/phosphonate transport system, was under positive selection. Bacteria usually uptake of inorganic phosphate by the high-affinity ABC-transport system Pst and Phn [92]. Moreover, previous research suggested that Phn possessed more than one physiological roles, namely perhaps transport of other phosphorus-containing molecules [93].

Cell division protein (ZapA) is one of the bacterial tubulin homolog FtsZ stabilisers. ZapA is widely conserved among bacteria with apparent orthologs in many species [94], and it was identified under positive selection in *A. ferrooxidans* in this study (Table S5 and Fig. 5c). Previous results suggested that ZapA deletion strain *Bacillus subitilis* had serious defects in bacterial division if it was knocked out together with the EzrA gene [94]. Meanwhile, ZapA has accessory roles in regulating pneumococcal physiology [95]. Additionally, OGs related to information storage and processing were identified under positive selection, such as HolC and HemK (Table S5 and Fig. 5d). DNA polymerase III, chi subunit HolC is involved in DNA replication and is the only protein of the holoenzyme that bound to single-strand DNA binding protein (SSB) [96]. HemK plays a role in mRNA translational termination. Previous research found a hemK knockout strain in *E. coli* suffered severe growth defects [97]. The three proteins of ZapA, HolC and HemK were related to cell growth, based on which, *A. ferrooxidans* may be able to better adapt to the environment by positive selection acting on the growth related factors. Peptidyl-prolyl isomerase (SurA), one of the known proline isomerases, was also under positive selection. SurA is essential for maintaining the integrity of bacterial membranes among Gram-negative bacteria and is a line of defence for survival in harsh environments [98]. Previous study has shown the members of *Acidithiobacillus* were remarkably adaptable owing to their ability to develop specialized mechanisms to cope with extremely harsh conditions [14]. Therefore, these genes seem to experience adaptive selection in *A. ferrooxidans* lineage.

### Interspecific divergences of Rus and T4SS

**Fig. 6.**
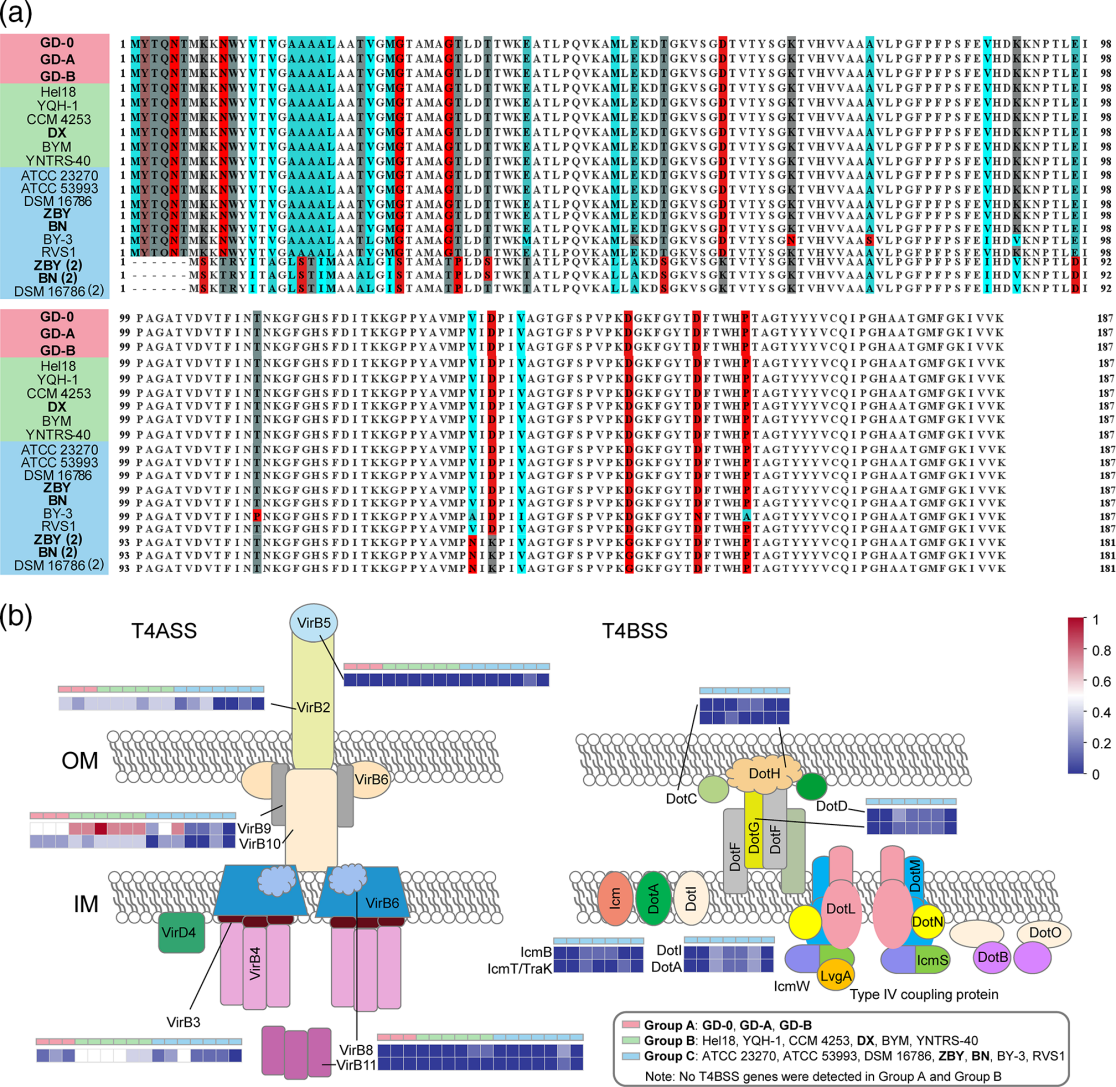
(**a**) Multiple sequence alignment among the Rus sequences. The differences were marked in colours. (**b**) T4SS-related genes in 16 *A *. *ferrooxidans* strains, the colour of heat map were correlated with the number of genes.

Rus, a soluble protein, can appear blue due to the combination of copper ions. It can transmit electrons from cyc2 to the cytochrome *aa_3_* complex or cytochrome *bc1* complex, and these two complexes couple with cytochrome *c4* protein (cyt *c552*, cyc1) in the downhill pathway and uphill way respectively [2, 99]. We found 19 Rus sequences from *A. ferrooxidans* genomes and conducted multiple sequence alignment among them (Fig. 6a). Each strain contained at least one Rus sequence encoded by *rusA*, while strains ZBY, BN and DSM 16786, which belonged to group C, contained two Rus sequences (the other one encoded by *rusB*). The Rus protein contains copper binding site, where a copper ion plays an essential role in stabilizing the protein [100]. Previous transcriptional experiment has shown that the expression of genes coding for the cytoplasmic CopZ-like copper-binding chaperone and the periplasmic copper-binding proteins Rus and AcoP, which was a part of the iron-oxidizing supercomplex, was increased when *A. ferrooxidans* was grown in the presence of copper [101]. Meanwhile, the intrinsic flexibility of the RusB protein could prevent *Acidithiobacillus* from losing its conducting capacity, that means *rusB* has better intrinsic flexibility than *rusA* [102, 103]. Therefore, we speculated that the existence of two Rus sequences in genomes may be related to the ore type in the environment, and was intended to help the strains better participate in iron metabolism. Overall, the RusA (except BY-3) and RusB protein was highly conserved during the evolutionary process [13].

T4SS, the most versatile secretion system, delivers proteins and nucleoprotein complexes into targeted cells. T4SS plays an essential role in determining bacterial genome plasticity and diversity by mediating conjugations. Currently, research on T4SS-mediated gene transfer mainly focused on antibiotic resistance gene transfer [101, 104]. We annotated the genes involved in T4SS among 16 *A *. *ferrooxidans* strains. The results showed that all strains had an incomplete T4SS in their genome except RVS1, but the types and numbers of T4SS-related genes contained in each strain were quite varied (Fig. 6b, detailed information about gene locations was in Table S6). ZBY and BN have the same T4SS composition as well as gene number, which was entirely consistent with the classification according to their collected locations (Fig. 1). So we proposed that the T4SS composition among *A. ferrooxidans* correlated with their geographical distribution. VirB2, VirB3, VirB9 and VirB10 were found in all *A. ferrooxidans* strains except ZBY, BN and RVS1. VirB2 is the major flagellin, VirB3 belongs to the inner membrane complex (IMC), and VirB9 and VirB10 belong to the outer membrane core complex (OMCC). The entire ATPase energy centre plus the IMC and OMCC sub-assemblies, but not the extended pilus, were required for substrate transfer, but we did not annotate the T4SS associated with ATPase [105].

T4ASS transporters contain P- and F- conjugative pilus, while the T4BSS transporters evolved from I-type conjugation systems [106]. ZBY, BN, DSM 16786 and BY-3 contained T4ASS and T4BSS (Fig. 6b), they all belonged to group C, indicating that T4SS in strains of group C had higher diversity and evolved differently from that of group A and B. Additionally, previous study discovered that genes of the T4SS were activated in response to antimonite (Sb(III)), inhibiting T4SS activity in *Bosea* sp. AS-1 dramatically reduced its oxidation efficiency and tolerance to Sb(III), establishing the T4SS as an important Sb resistance factor in bacteria [107], so T4SS was also implicated in microbial metal resistance. It can be seen that the diversity of T4SS in group C was likely to help the strains adapt to the extreme environments.

## Conclusion remarks

In this study, we performed a comparative genomic analysis of 16 *A *. *ferrooxidans* genomes, including six newly sequenced strains isolated from China and Zambia (strains GD-0, GD-A, GD-B, DX, ZBY and BN). The results indicated that *A. ferrooxidans* diverged into three groups from a common ancestor. The *A. ferrooxidans* species presented as an ‘open’ pan-genome, suggesting the addition of new genomes would expand their gene repertoire. Furthermore, some gene functions related to environmental adaptation were obtained at the key nodes during the evolutionary process. In the key nodes of *A. ferrooxidans* evolutionary process, a number of MGEs were gained or lost, indicating that HGTs frequently occurred during the evolution. In contrast, the genomes of group A strains were smaller. Both gene gain and loss can promote environmental adaptation in the process of *A. ferrooxidans* pedigree evolution. This finding also showed the prevalence of gene loss coupled with genome streamlining. In other words, the fitness cost of improving environmental adaptation might drive the evolution of *A. ferrooxidans* genomes. Positive selection analysis of 16 *A *. *ferrooxidans* showed that OGs of dN/dS>1 were mainly involved in metabolism, especially COG category [H] coenzyme transport and metabolism. These OGs seemed to have experienced adaptive selection in *A. ferrooxidans* lineages. We also found a large diversity of Rus sequences and T4SS composition among *A. ferrooxidans* strains, especially group C, which was likely to be related to the geographical differentiation.

In summary, genetic communication through exchange of genetic materials contributed to shaping the genomes of *A. ferrooxidans *. We further proposed that microorganisms originating under the same conditions can adapt to extreme environments after acquisition of multiple adaptive functions, and then evolve into different species. Taken together, the findings provided evidences for the relatedness between the hereditary variation of *A. ferrooxidans* genomes with their adaptive evolution, and provided theoretical support for the understanding of *Acidithiobacillus* in extreme environments.

## Supplementary Data

Supplementary material 1

Supplementary material 2

Supplementary material 3

Supplementary material 4

Supplementary material 5

Supplementary material 6

Supplementary material 7
