# Supplementary material for: Comparative genomics reveals intraspecific divergence of Acidithiobacillus ferrooxidans: insights from evolutionary adaptation
Source: Microb Genom. 2023 Jun 7;9(6):mgen001038. doi: 10.1099/mgen.0.001038 (PMC10327505; doi:10.1099/mgen.0.001038)

Supplementary information

Comparative genomics reveals intraspecific divergence of *Acidithiobacillus ferrooxidans*: insights from evolutionary adaptation

Rui Liu [a], Liyuan Ma [a,b,e*], Hongmei Wang [a,b,c], Deng Liu [a,b,c], Xiaolu Lu [a,b], Xinping Huang [a], Shanshan Huang [d], Xueduan Liu [d]

[a] *Hubei Key Laboratory of Yangtze Catchment Environmental Aquatic Science, School of Environmental Studies, China University of Geosciences, Wuhan 430074, China*

[b] *Hubei Key Laboratory of Wetland Evolution & Ecological Restoration, School of Environmental Studies, China University of Geosciences, Wuhan 430074, China*

[c] *State Key Laboratory of Biogeology and Environmental Geology, China University of Geosciences, Wuhan 430074, China*

[d] *School of Minerals Processing and Bioengineering, Central South University, Changsha 410083, China*

[e] *School of Engineering, Cardiff University, Cardiff CF243AA, United Kingdom*

***Corresponding author at:** School of Environmental Studies, China University of Geosciences, Wuhan 430074, China. *E-mail address*: maly@cug.edu.cn (L. Ma), MaL28@cardiff.ac.uk

Fig. S1 Phylogenetic tree of 16S rRNA genes showing the relationships between newly sequenced strains and other *Acidithiobacillus* strains. *Acidiphilium multivorum* was an outgroup. For some strains, 16S rRNA gene sequences were extracted from the complete or draft genome using RNAmmer.

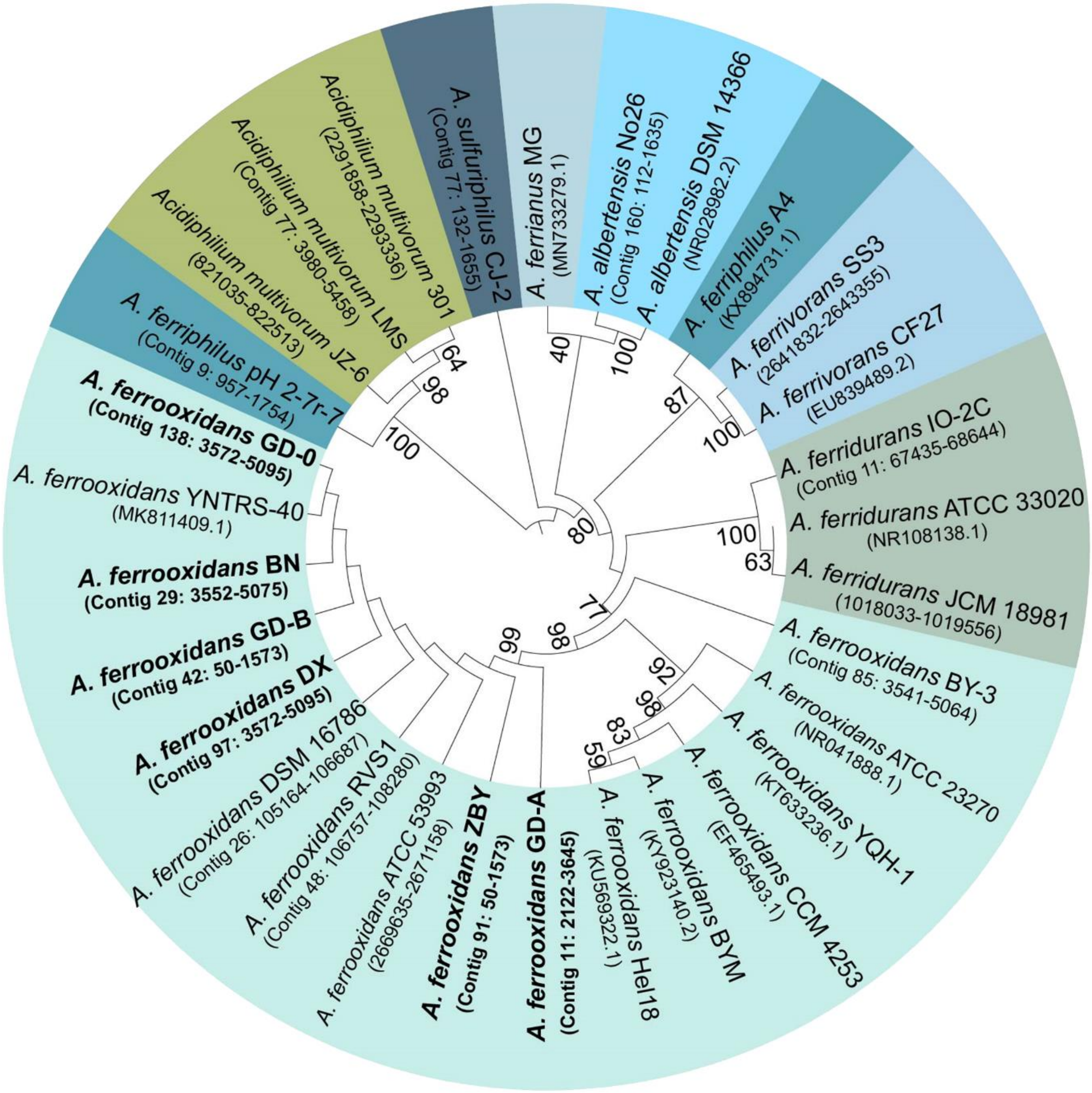

Fig. S2 Phylogenetic tree of 16S rRNA genes showing the relationships between *A. ferrooxidans* and other acidic chemolithoautotrophs. Bootstraps values ≥70% were represented by black dots on the nodes.

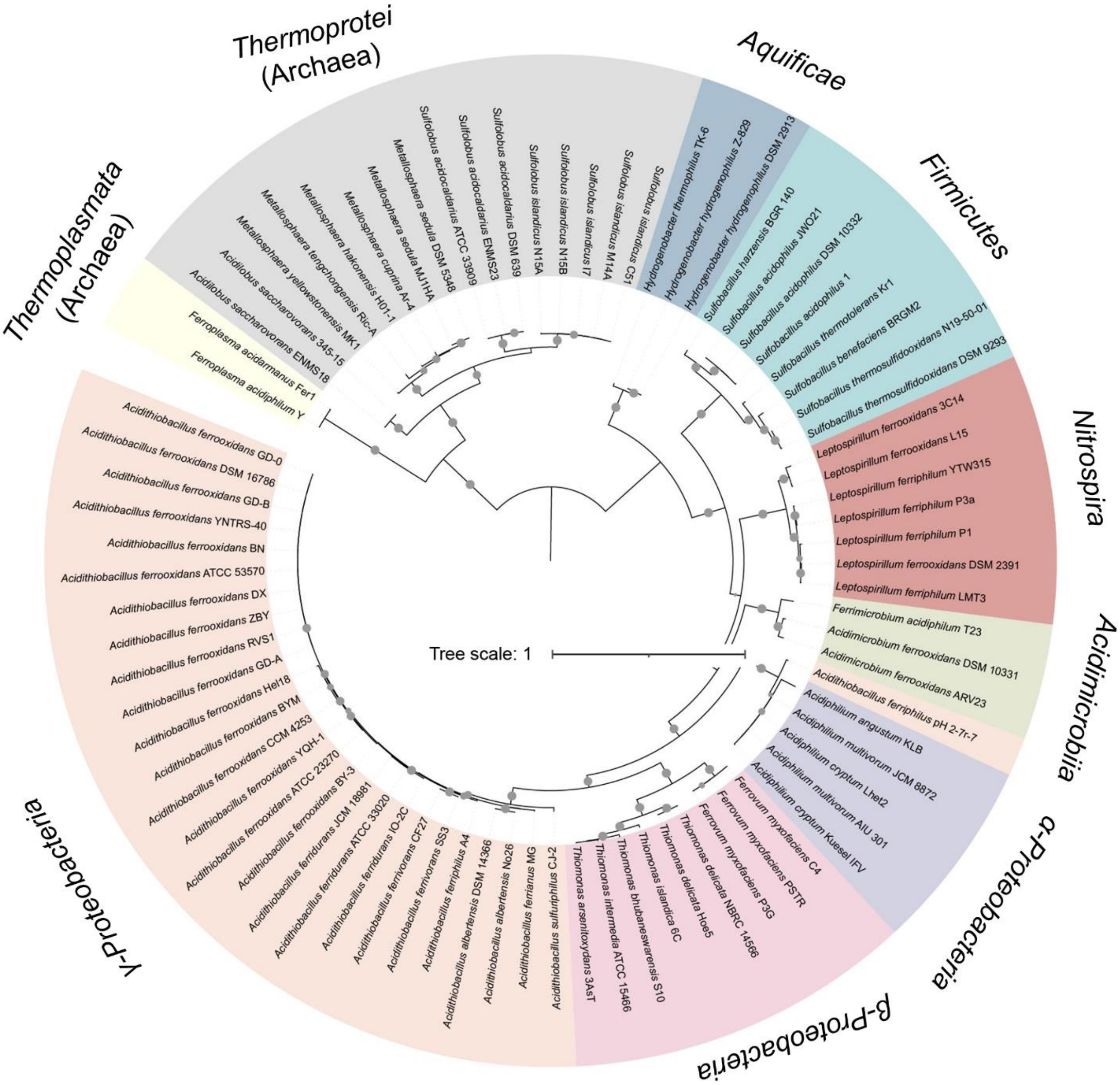

Fig. S3 The COG functional assignment of genes from six *A. ferrooxidans* strains.

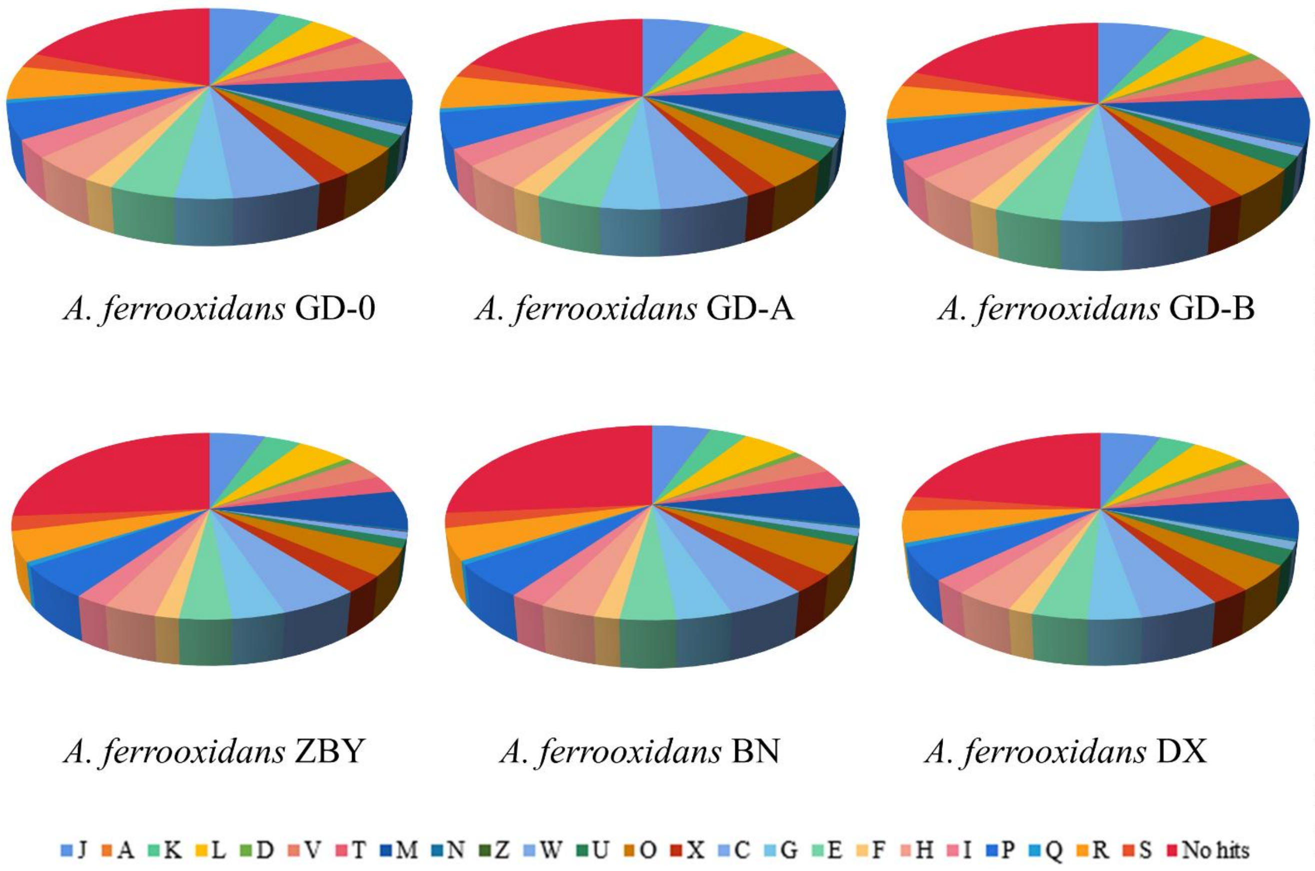

Information storage and processing

[J] Translation, ribosomal structure & biogenesis
[A] RNA processing and modification
[K] Transcription
[L] Replication, recombination & repair

Cellular processes and signaling

[D] Cell cycle control, cell division, chromosome partitioning
[V] Defense mechanisms
[T] Signal transduction mechanisms
[M] Cell wall/membrane/envelope biogenesis
[N] Cell motility
[Z] Cytoskeleton
[W] Extracellular structures
[U] Intracellular trafficking, secretion & vesicular transport
[O] Post-translational modification, protein turnover & chaperones

Mobilome

[X] Mobilome: prophages, transposons

Metabolism

[C] Energy production & conversion
[G] Carbohydrate transport & metabolism
[E] Amino acid transport & metabolism
[F] Nucleotide transport & metabolism
[H] Coenzyme transport & metabolism
[I] Lipid transport & metabolism
[P] Inorganic ion transport & metabolism
[Q] Secondary metabolites biosynthesis, transport & catabolism

Poorly characterized

[R] General function prediction only
[S] Function unknown

Fig. S4 (A) Pan-Genome analysis among BY-3, ATCC 23270 and ATCC 53993, (B) six newly sequenced strains with two type *A. ferrooxidans* strains, and (C) six newly sequenced *A. ferrooxidans* strains.

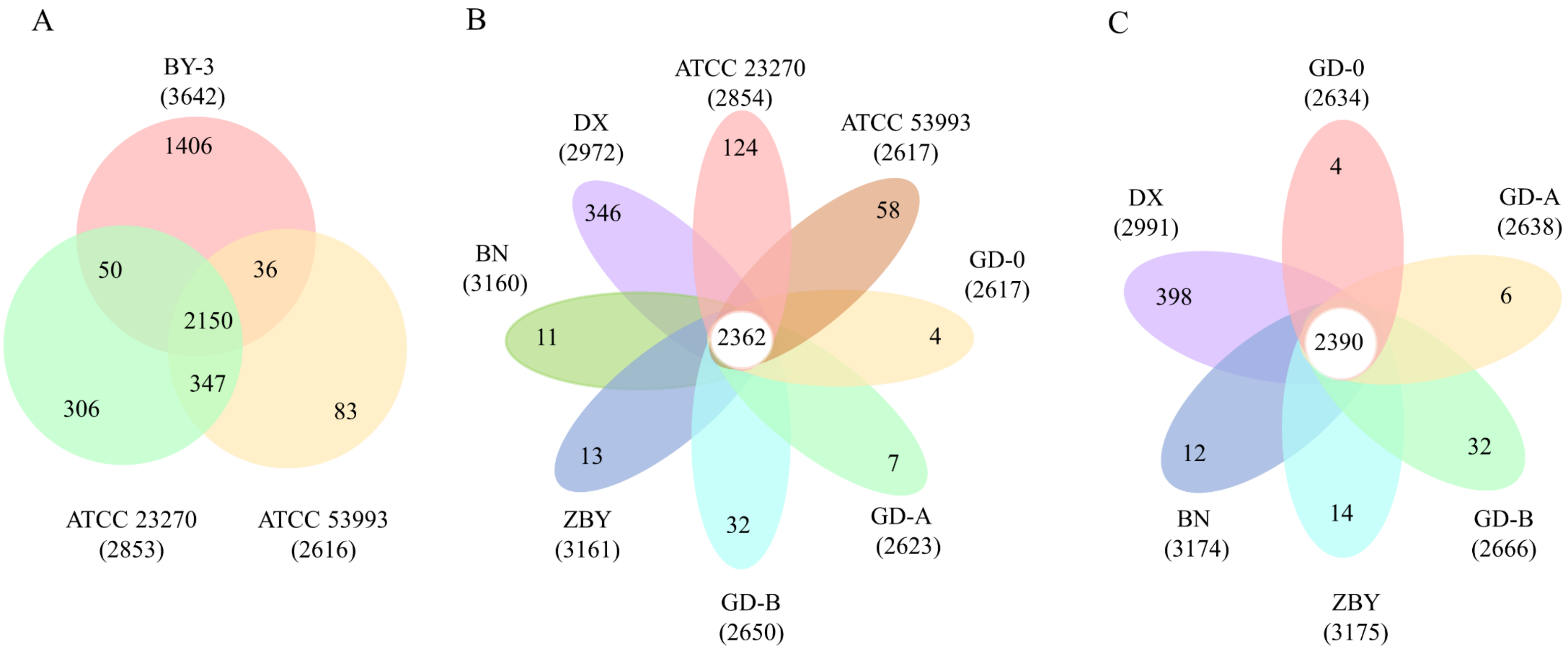

Fig. S5 Pan-genome and core genome evolution of *A. ferrooxidans*.

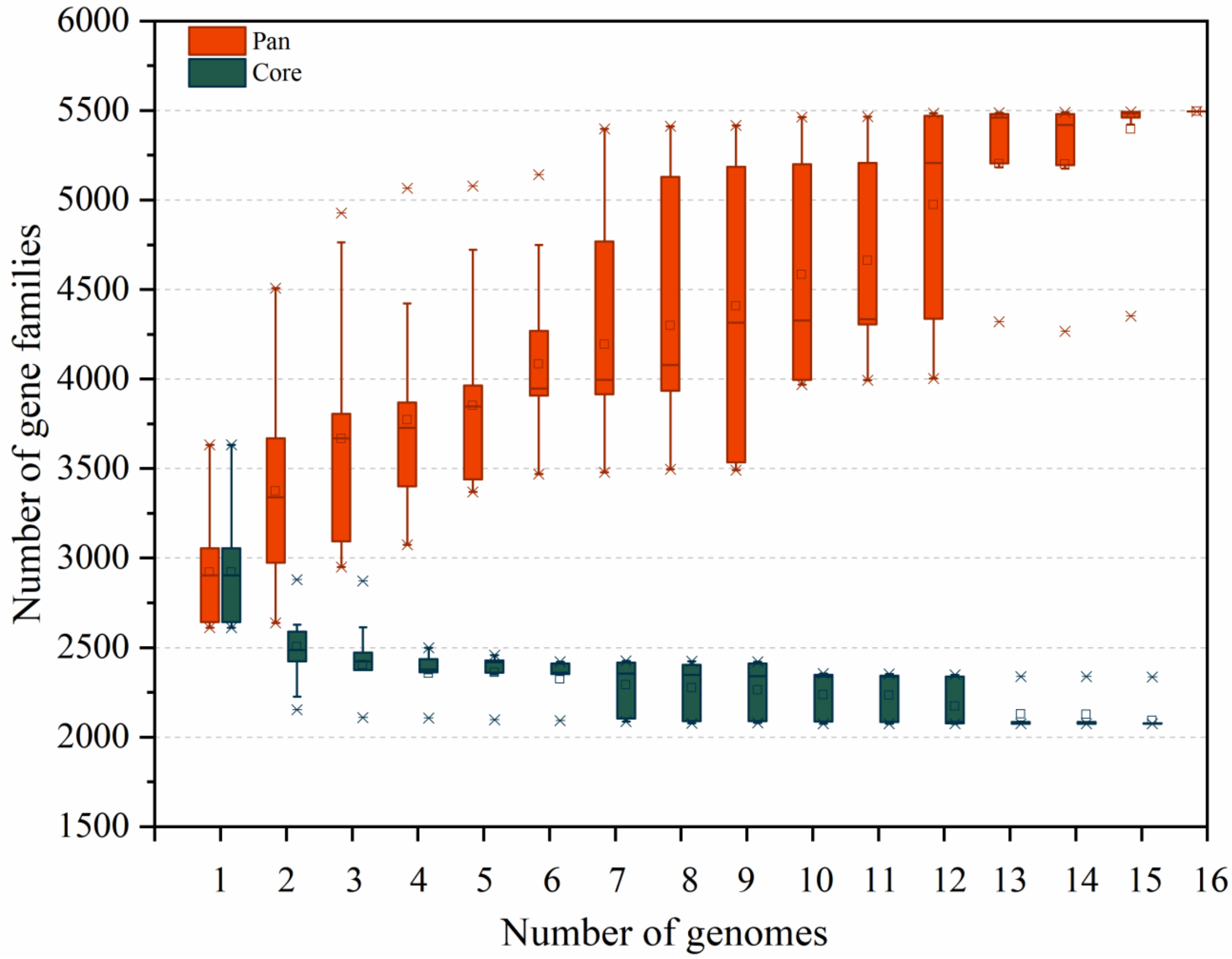

Supplement: Supplementary material 1 [file mgen-9-1038-s001.pdf]
